# Monte carlo study of detection limit for gold nanoparticles within small animal sized object during benchtop X-ray fluorescence computed tomography

**DOI:** 10.1038/s41598-026-54278-6

**Published:** 2026-05-27

**Authors:** Neerajan Nepal, Sandun Jayarathna, Sang Hyun Cho

**Affiliations:** 1 Department of Radiation Physics, The University of Texas MD Anderson Cancer Center, Houston, TX 77030 USA; 2 Department of Imaging Physics, The University of Texas MD Anderson Cancer Center, Houston, TX 77030 USA

**Keywords:** X-ray fluorescence, Benchtop x-ray fluorescence computed tomography, Pixelated cadmium telluride detector, Gold nanoparticles, Materials science, Nanoscience and technology, Physics

## Abstract

This Monte Carlo (MC) study investigated theoretically achievable detection limits of gold nanoparticles (GNPs) present within a small-animal-sized phantom during benchtop x-ray fluorescence (XRF) computed tomography (XFCT) imaging based on the detection of gold K-shell XRF photons emitted from GNPs. The MC model was based on our experimental benchtop cone-beam XFCT system, reflecting an anticipated upgrade adopting a larger field-of-view pixelated cadmium-telluride detector that fully covers a 3 cm-diameter PMMA phantom including three 6 mm-diameter cylindrical holes filled with various concentrations of GNPs. It was validated by comparing the MC results with experimental data acquired under identical conditions using a 1.8 mm-Sn-filtered 125 kVp beam. The MC simulations were then extended to investigate GNP detection limits across a range of x-ray doses, GNP concentrations, and incident x-ray beam spectra. Assuming an ideal detector, the GNP detection limits of 104–140 ppm at 1.775 cGy and 69–88 ppm at 3.55 cGy were achievable with the 1.8 mm Sn-filtered 125 kVp beam. When hypothetical monochromatic beams with their energy above the gold K-edge were employed, the GNP detection limits were improved by up to three-folds at a given dose (e.g., 34–70 ppm at 1.775 cGy with the 85 keV beam).

## Introduction

X-ray fluorescence (XRF) computed tomography (XFCT) is an imaging technique that identifies and quantifies elemental composition within an imaging object by detecting characteristic x-rays (or XRF photons) emitted from it when irradiated by an x-ray source^1–7^. As each element emits XRF photons at unique energy, an XFCT system provides element-specific contrast, offering distinct advantages over conventional attenuation-based x-ray imaging (e.g., transmission x-ray CT)^8^. For example, since its inception in 1980s, an original form of XFCT (inclusive of direct XRF imaging/mapping), developed with monochromatic/synchrotron x-ray sources at synchrotron facilities, has successfully been applied for quantitative imaging of both exogenous and endogenous metallic elements including iodine-based agents and gold nanoparticles (GNPs) present within biological samples and imaging objects (e.g., small animals)^3,4,9–11^. Additionally, over the past 15 years or so, another form of XFCT, implemented on a benchtop setting using ordinary polychromatic x-ray sources (typically known as benchtop XFCT)^12^, has emerged as a promising modality for preclinical quantitative imaging of high atomic number (Z > 50) metal nanoparticles (MNPs) (e.g., GNPs^8,13–15^ and gadolinium NPs^16,17)^ as well as other MNPs or metal probes made of low-Z elements (e.g., molybdenum^18,19^ and ruthenium^20)^. Unlike quantitative imaging studies performed with conventional nuclear imaging modalities available at typical biomedical laboratories such as positron emission tomography (PET) and single photon emission CT (SPECT), these benchtop XFCT imaging studies did not require radiotracers, possibly alleviating radiation safety related hassles^12^. Other advantages of benchtop XFCT as suggested previously^8^ are also discernable from these studies, for example, its capability for tomographic imaging of larger objects and longitudinal molecular imaging studies, compared to optical imaging and hyperpolarized magnetic resonance imaging (MRI), respectively.

For preclinical quantitative imaging with MNPs such as GNPs that have been actively investigated for cancer molecular imaging and radiation therapy applications^21–30^, the primary objective of benchtop XFCT is to determine the biodistribution of either passively targeted (i.e., unconjugated) or actively targeted (e.g., conjugated with tumor-targeting moieties) MNPs present in small animals. While it was commonly pursued by the majority of the aforementioned benchtop imaging studies, this objective could not be achieved by a universal means involving a particular benchtop XFCT system and technique as each of the quantitative imaging tasks poses unique challenges. For example, the benchtop XFCT systems used in the aforementioned studies were different from one another, in terms of the incident x-ray source energy/spectrum (e.g., quasi-monochromatic beams below 40 keV energy vs. polychromatic beams above 80 keV average energy), the incident x-ray beam geometry (e.g., cone vs. pencil), the XRF photon detection geometry (e.g., pinhole vs. parallel hole), the XRF detector (e.g., silicon drift detector vs. cadmium telluride detector), the MNPs that need to be imaged (e.g., low-Z vs. high-Z MNPs), and some practical aspects (e.g., building cost and translational potential of the system for widespread uses). Thus, an accurate comparison of the capabilities (e.g., pros vs. cons) among the various benchtop XFCT systems requires rather careful consideration of all these aspects. In general, within the context of the whole body imaging of small animals injected with MNPs, the benchtop XFCT systems used in the aforementioned MNP-based imaging studies appear to provide a wide range of system sensitivity (material detection limit), total x-ray dose and scan time, with the reported values of ~ 50 to 500 parts-per-million (ppm), ~ 1 cGy to 1 Gy, and ~ 10 min to 1 h, respectively. Despite various limitations related to the technical specifications listed here, these benchtop XFCT systems appear to have enabled the in vivo whole body imaging of small animals injected with MNPs.

In the case of GNPs, gold (Z = 79), the base metal, emits relatively high energy K-shell XRF photons (~ 67–69 keV) that are capable of penetrating through small animal-sized objects consisted of water/tissue-like media, when irradiated with an incident x-ray beam containing photons with their energy above the gold K-edge (80.7 keV). Thus, this specific mode of benchtop XFCT utilizing gold K-shell XRF photons, often referred to as K-shell XFCT (vs. L-shell XFCT that utilizes ~ 10 keV gold L-shell XRF photons), has been the subject of active investigation for the whole body imaging of small animals injected with GNPs^7,8,31^. The main challenge of K-shell XFCT stems from the use of a polychromatic x-ray beam for excitation of XRF photons which causes inherently weak XRF photon yield from GNPs and substantial Compton-scattered photon background, obscuring the gold K-shell XRF photon peaks^12,31,32^. Most notably, these aspects increase difficulty in extracting gold K-shell XRF signal as the concentration of GNPs present in an imaging object decreases. Thus, research efforts on K-shell XFCT typically focus on overcoming such difficulty through optimization of a polychromatic incident x-ray beam spectrum and hardware components associated with the x-ray beam production as well as XRF detection. As pointed out elsewhere^30^, however, a threshold for GNP (i.e., gold) detection limit may exist for K-shell XFCT, given the fundamental laws of physics governing the operation of K-shell XFCT. Thus, it is desirable to identify such a threshold through computational and/or experimental investigations so that future research effort on K-shell XFCT will be steered into the right direction.

In general, the specific value of GNP detection limit (per given x-ray dose) can vary, depending on the design of a K-shell XFCT system (affecting gold XRF production and detection), the material/size of GNP-containing object, and the experimental conditions (or computational models reflecting such conditions). Accordingly, there is practical difficulty in deriving the value or a range of values for the GNP detection limit that can be universally associated with K-shell XFCT. Recognizing this difficulty, therefore, the current work focused on investigating the GNP detection limit achievable from a well-documented K-shell XFCT system such as the K-shell XFCT mode of our latest benchtop cone-beam XFCT system^6,7^. To facilitate this investigation under the varying conditions (in terms of GNP concentration, x-ray dose, and incident beam spectrum) in a consistent manner, we developed a new Monte Carlo (MC) model of our benchtop XFCT system, based on our original experimentally validated MC model^33^. Through this new MC model, we also intended to project the GNP detection limit that can be achievable from an upgraded system providing a field of view (FOV) large enough to cover small animal-sized objects (e.g., 3 cm diameter GNP-containing phantom), compared to our latest system providing a smaller FOV (2 cm x 2 cm). In principle, this particular upgrade would be straightforward, if not restricted financially, given the feasibility of developing a custom 2 × 2 array detector based on a smaller FOV detector system within our existing benchtop XFCT system and the commercial availability of a 2 × 2 array detector system based on the same smaller FOV detector system^34^. However, it will require a redesign of our detector collimator and thus may affect the efficiency of XRF detection, hence the GNP detection limit. Thus, the current MC study provided an important outlook regarding the GNP detection limit (e.g., whether or not it will change) after the anticipated detector system upgrade.

In essence, the present MC study served for the two primary purposes. First, as noted above, it allowed for the projection of GNP detection limit under the K-shell XFCT mode of our benchtop XFCT system upgraded with a larger FOV detector. According to the results from our recent experimental study^7^(as updated following the correction^35^, our latest benchtop XFCT system with a smaller FOV detector system was capable of detecting/imaging GNPs in a dose dependent manner, i.e., as low as 500 ppm (or 0.05 wt%) at 1.775 cGy per projection (delivered over 5 s using a 1.8 mm Sn-filtered 125 kVp beam) and 300 ppm (or 0.03 wt%) at 3.55 cGy per projection (delivered over 10 s using the same Sn-filtered 125 kVp beam). Within the context of small animal imaging, these results suggest that our latest benchtop XFCT system allows for the imaging (with FOV of 2 cm x 2 cm) of a mouse containing 300–500 ppm GNPs with the total x-ray dose of 0.5–1 Gy and the scan time much less than 10 min (with 30 projections). Thus, this benchtop XFCT system closely meets the minimum technical requirements as suggested for the whole body imaging of live mice injected with GNPs^30^. In the current MC study, we attempted not only to investigate the validity of these findings despite the anticipated detector upgrade but also to conduct a more thorough study on the GNP detection limit by taking advantage of greater flexibility in adjusting various parameters in question (e.g., GNP concentration, phantom irradiation setup, and incident x-ray beam spectrum). As a result, we were able to determine the GNP detection limits that may be considered as theoretically the lowest (i.e., threshold) values under the given conditions.

Besides, the aforementioned flexibility through our MC model allowed us to achieve the second goal of the current study, which was to investigate the GNP detection limits achievable from the adoption of hypothetical monochromatic x-ray beams with their energy just above the gold K-edge (i.e., 85, 90, and 95 keV) for potentially optimized gold XRF photon production/detection during K-shell XFCT. This investigation was particularly noteworthy, given the previous studies^36,37^ that suggested the possibility of drastic improvement (by up to an order of magnitude) in the GNP detection limit for K-shell XFCT by adopting such monochromatic x-ray beams as considered in this study. Overall, the current MC study provided valuable insight into theoretically projected limitations of K-shell XFCT, in terms of detecting/imaging GNPs present at low concentration (on the order of 100 ppm or lower) within small animals or similarly sized objects consisted of water/tissue-like media.

## Methods

### Monte Carlo model

**Fig. 1:**
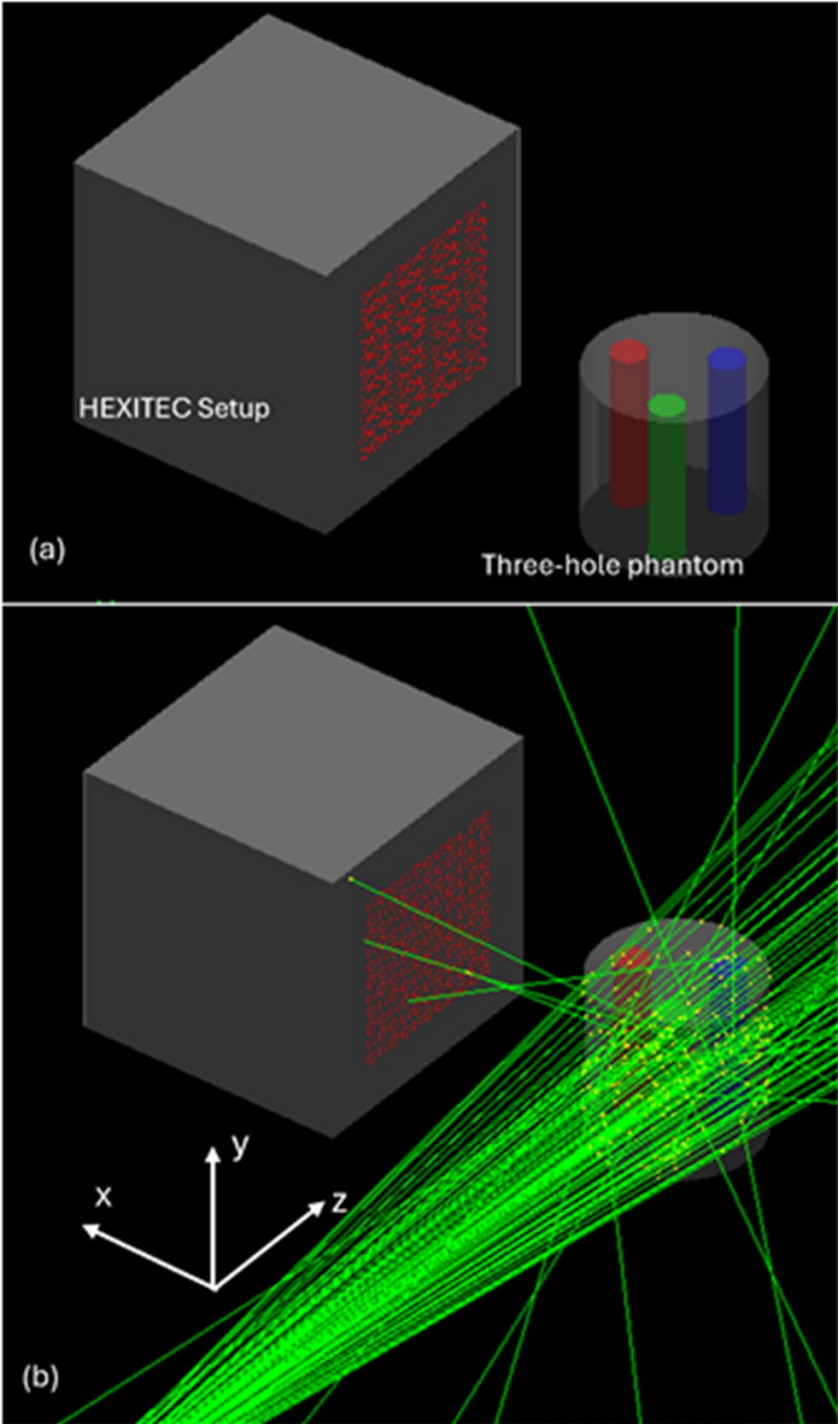
(**a**) Geant4 model of a 3 cm-diameter three-hole (6 mm-diameter each) imaging phantom, (**b**) Illustration showing the GNP-containing three-hole phantom irradiated by a cone-beam incident along the z-direction. This figure shows a pixelated CdTe detector (modeled based on a commercial “HEXITEC” detector system with an ideal FOV of 3 cm x 3 cm) and a three-hole phantom within an experimental benchtop cone-beam XFCT system. In the HEXITEC setup, a pixelated CdTe detector is coupled with a 5 cm-thick 2-dimensional parallel hole collimator made of stainless steel including an 11 × 11 array of 2 mm-diameter apertures (as shown by red colored marks). Each row of collimators corresponds to each axial slice of the object to be imaged.

An MC model of our experimental benchtop cone-beam XFCT system^7^ was developed using the Geant4 MC simulation toolkit (release 11.0.0)^38–41^ as shown in Fig. 1. The model consisted of a 3 cm diameter PMMA phantom including three 6 mm diameter cylindrical holes (“three-hole phantom”) and a pixelated cadmium telluride (CdTe) detector including 128 × 128 pixels (pixel pitch of 250 μm). While based on a commercially available HEXITEC detector system (Quantum Detectors, Oxford, UK) with a 2 cm x 2 cm CdTe sensor (providing a 2 cm x 2 cm FOV)^42^, the CdTe detector model in the current MC study was an ideal one with a larger CdTe sensor or FOV of 3 cm x 3 cm so that the detector translation was not needed to cover the entire 3 cm diameter phantom. The CdTe detector was coupled with a 5 cm thick 2-dimensional (2D) parallel hole collimator made of stainless steel including an 11 × 11 array of 2 mm-diameter apertures covering the 3 cm × 3 cm FOV. The distance between the x-ray source and the isocenter was fixed at 15 cm and the distance between the isocenter and the detector was at 10 cm, mirroring our experimental setup^6,7^. The energy of XRF/scatter photons reaching the CdTe detector was scored during the simulation with an energy bin size of 0.1 keV, reflecting an ideal detection scenario to facilitate theoretical analysis.

The current MC work considered two different types of incident x-ray sources – a realistic polychromatic x-ray source as adopted in our experimental XFCT setup and hypothetical monochromatic x-ray sources for theoretical analysis and comparison. Specifically, two polychromatic 125 kVp x-ray source spectra filtered with 1.8 mm thick tin (Sn) were employed in MC simulations for different purposes. The first Sn-filtered 125 kVp spectrum was generated using SpekPy package (V2.0) in Python^43,44^, and used for MC model validation and GNP detection limit estimation. For MC simulations of K-shell XFCT imaging with our GNP-filled three-hole phantom, we used the second Sn-filtered 125 kVp spectrum that was almost identical to the first spectrum but based on our original experimentally-validated Geant4-based MC model^33^, in order to maintain some consistency in our K-shell XFCT imaging simulations so that, if necessary, the imaging performance under different conditions (or between our MC studies) could be directly compared. For GNP detection limit estimation for hypothetical monochromatic x-ray sources, monochromatic x-ray beams with varying energy above the gold K-absorption edge (i.e., 85, 90, and 95 keV) were used. In all cases, the x-ray beam was cone-shaped, with a diameter of 3 cm at the isocenter. For K-shell XFCT imaging of a three-hole phantom loaded with varying concentrations of GNPs, the Sn-filtered 125 kVp beam in our experimental cone-beam XFCT setup delivered the dose of 3.55 cGy per projection (in 10 s) or 1.775 cGy per projection (in 5 s)^6,7^. In this study, therefore, the two dose levels of 1.775 and 3.55 cGy were used as reference values for analysis, and the corresponding numbers of incident x-ray photons required to achieve these dose levels were determined as shown in Table 1. Notably, all monochromatic x-ray beams required higher numbers of photons compared to the Sn-filtered 125 kVp beam to deliver a given dose.

**Table 1:** Total number of incident photons to deposit 1.775 and 3.55 cGy dose at the center of the three-hole phantom for different x-ray beam type.

Beam Type	Number of incident photons (× 10^9^) to deposit a given dose	
	1.775 cGy	3.55 cGy
Sn-filtered 125 kVp	650	1300
85 keV	770	1540
90 keV	720	1440
95 keV	675	1350

The MC model was validated quantitatively under the same XFCT imaging conditions as used in our previous experimental study^7,35^ to confirm its suitability for theoretical investigation of the GNP detection limit in this study. Quantitative spectral validation was carried out by comparing the MC results (i.e., simulated XRF/scatter spectrum) with experimental results acquired from 10 s irradiation (delivering 3.55 cGy) of a three-hole phantom containing 10,000 ppm (or 1 wt%) GNP solution in one of the holes using the Sn-filtered 125 kVp x-ray beam. For a proper comparison with experimental results, the spectral data acquired from MC-simulation were rearranged using an energy bin size (0.8 keV) closely matching a realistic detector resolution^6,33^. The agreement between MC-simulated and measured spectra was quantitatively assessed using the Pearson correlation coefficient, focusing on the gold K-shell XRF peaks and the Compton background profile around the XRF peaks.

After the validation of the MC model, the three-hole phantom was configured for the determination of GNP detection limits under the irradiation by different x-ray beams shown in Table 1. In this configuration, GNP solution (between 0 and 500 ppm concentration) was filled in only one hole within the phantom, while the other two holes had water only (i.e., no GNP solution). The GNP-filled hole was oriented towards the detector to represent the most favorable scenario (rotation angle 0^o^) and rotated 180 degrees for the least favorable, in order to investigate the effect of XRF photon attenuation within the phantom on the GNP detection limits.

Similar to MC simulations described above, the MC model was used for simulation of K-shell XFCT imaging under the same XFCT imaging conditions as used in our experimental work^7,35^ but with an ideal detector (i.e., providing an energy resolution of 0.1 keV), in order to assess the benchtop XFCT imaging capability under the most favorable but hypothetical imaging scenario. Initially, the three-hole phantom in the MC model was configured to include three cylindrical holes filled with GNPs at 1000, 3000, and 5000 ppm concentrations, respectively. This phantom was then rotated through 360 degrees in the step size of 12 degrees, resulting in a total of 30 projections during the XFCT imaging. The Sn-filtered 125 kVp beam was employed to deliver doses of 1.775 and 3.55 cGy per projection as in our experimental work^7,35^, resulting in 53.25 and 106.5 cGy of the total dose to the three-hole phantom, respectively. Subsequently, the GNP concentrations within the three-hole phantom were changed to 300 ppm – approximately at the threshold of detectability per our experimental studies^7,31,35^– in one of the holes, alongside 500 and 1000 ppm in the other two holes. MC simulations were then repeated in the same manner as described above.

The transport of photons and electrons was simulated using the Penelope low energy electromagnetic physics list in the Geant4 toolkit, with an energy range set from 100 eV to 200 keV. Photon transport mechanisms included Rayleigh scattering, photoelectric effect, and Compton scattering. The electron transport was modeled considering multiple scattering, Coulomb scattering, ionization, and bremsstrahlung x-ray production. Regardless of the material of passage, the production cut applied for photons and electrons were 1 mm and 1 μm, respectively. MC simulations were performed using the High-Performance Computing (HPC) cluster at The University of Texas MD Anderson Cancer Center in multi-thread mode. To simulate two billion incident photons, it took 128, 96, 105, and 119 min for the Sn-filtered 125 kVp, 85 keV, 90 keV and 95 keV beam, respectively. The scored energy spectra were saved in a CERN ROOT file format^45^ in the simulations.

**Fig. 2:**
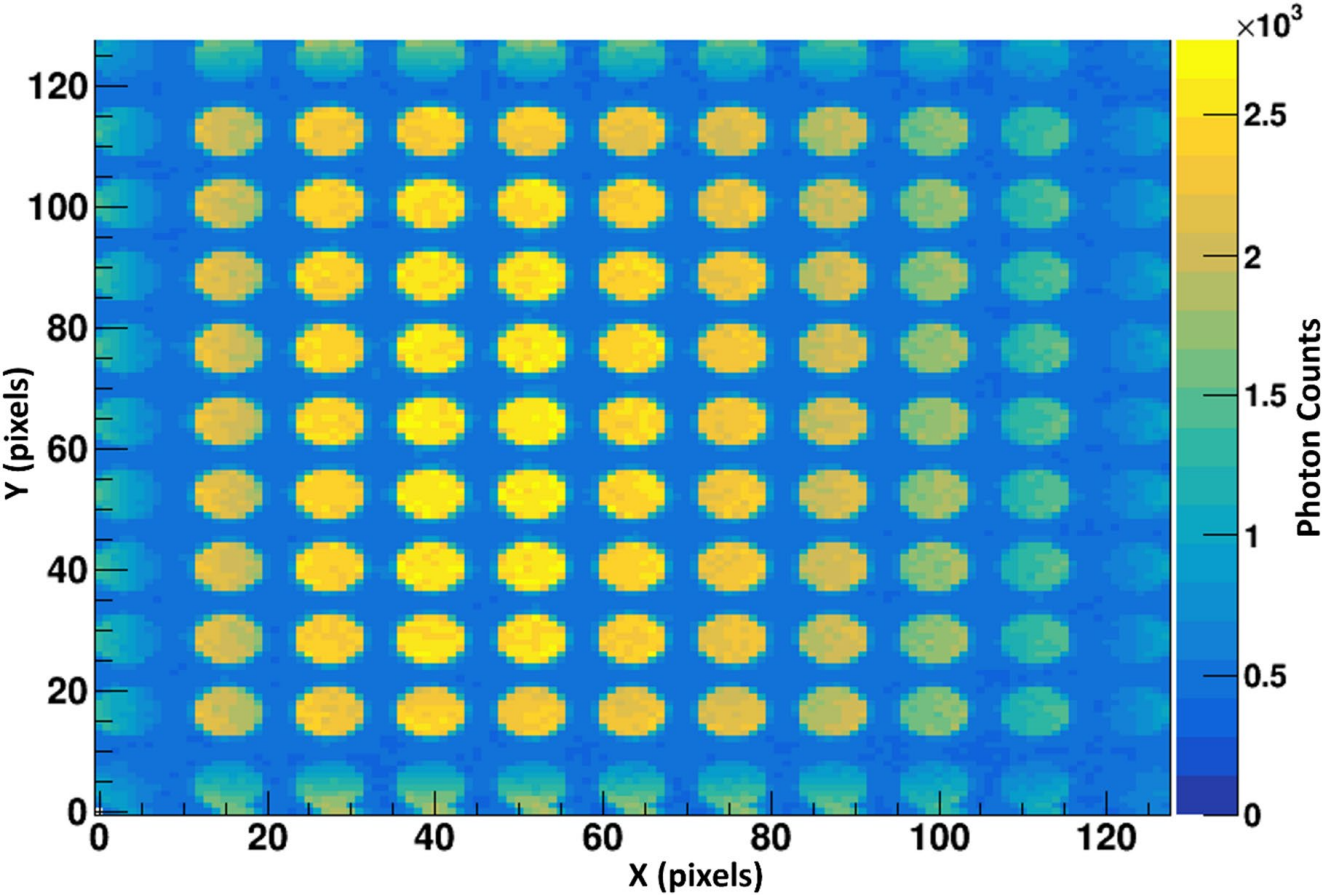
Two-dimensional (2D) image mimicking the detector sensor area. Each block/circular area corresponds to the area within the CdTe sensor created by the detector collimator aperture.

### Spectral data acquisition, processing, and analysis

#### XRF/scatter photon spectra acquisition

**Fig. 3:**
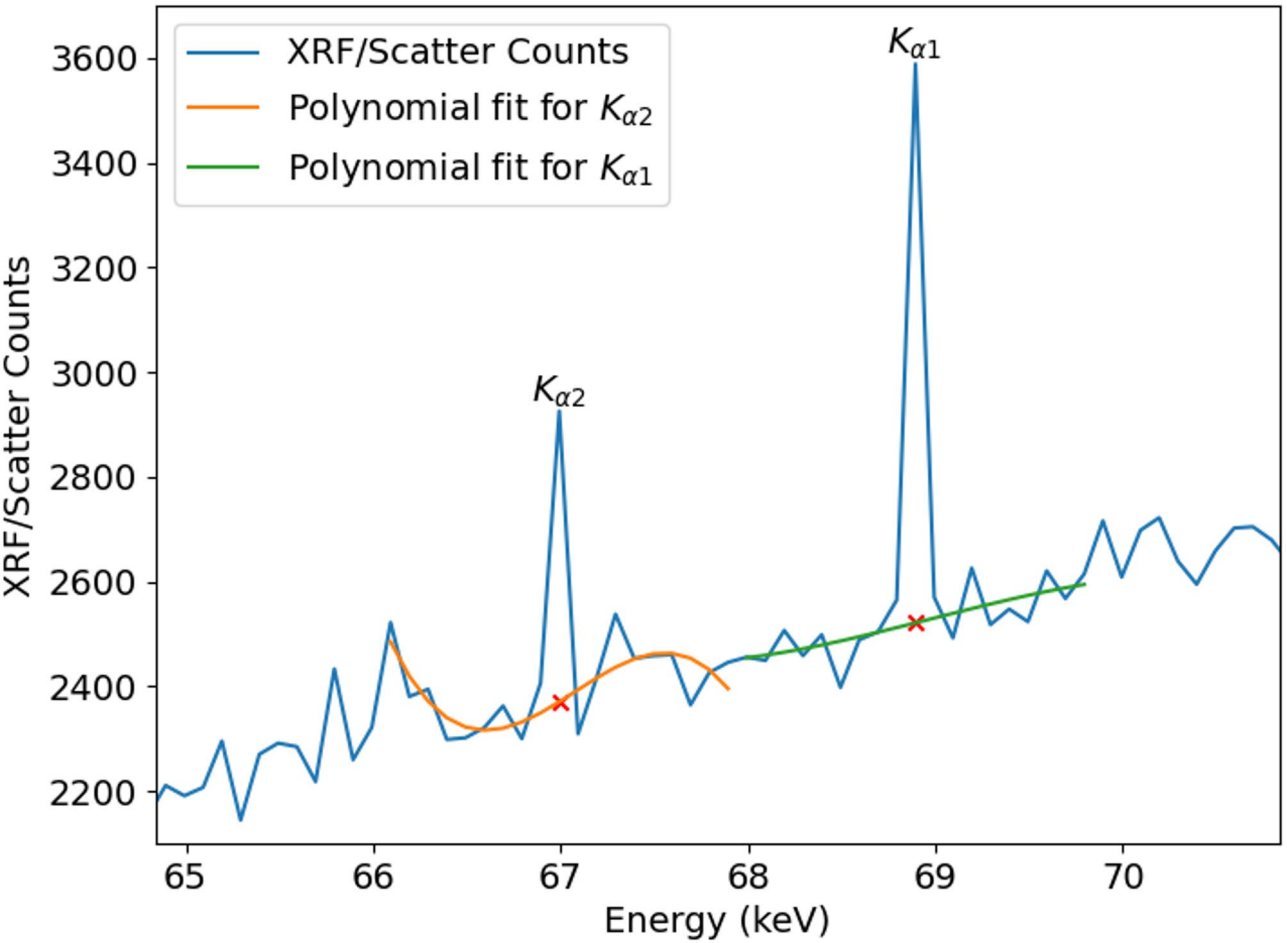
The XRF/scatter photon spectra scored from the nine vertically centered collimators corresponding to the pixelated detector due to irradiation of the three-hole phantom containing 500 ppm GNPs in one of the holes by the Sn-filtered 125 kVp beam depositing 3.55 cGy dose. The total area of two gold K-shell XRF peaks is measured by subtracting the corresponding background.

Figure 2 shows the 2D image acquired from the pixelated detector while depositing 3.55 cGy dose to the center of the three-hole phantom containing 500 ppm in one of the holes using the Sn-filtered 125 kVp beam. The 11 × 11 array of collimator holes is clearly visible across the entire detector or sensor area, where the color of each collimated area corresponds to the number of detected photons. The collimated areas, represented by high-intensity pixel clusters, are spatially well-separated, confirming the effectiveness of the detector collimator design. To enhance the spectral stability, energy spectra from individual pixels within each collimated area (defined over a 10 × 10-pixel area) were merged, producing composite spectra as shown in Fig. 3, following a pixel-by-pixel spectrum merging method developed in our prior studies^6,7^.

#### XRF signal extraction

The composite XRF/scatter spectra, generated from each projection, were analyzed by applying a polynomial fit (2nd order) within a ± 1 keV window centered on each gold XRF peaks, excluding the channels corresponding to each XRF peak region, to estimate the local spectral background as shown in Fig. 3. This approach effectively accounted for contributions from Compton scatter, allowing for an accurate isolation of the gold XRF peak. The total XRF photon counts associated with a peak was then obtained by subtracting the fitted background from the measured spectral counts. As in our previous studies^6–8,31^, a peak was considered a true signal only if its counts exceeded 1.96 times the standard deviation of the background, corresponding to a 95% confidence level ensuring the statistical reliability.

#### Attenuation correction

Compton scatter-based attenuation correction method, as used in our previous studies^31,46–48^, was also applied in this work. Briefly, the background subtracted XRF signal was normalized using the Compton scatter peak to correct for the attenuation of both the incident x-ray beam and XRF photons emitted from GNPs.

#### Calibration curves and detection limit

The average XRF signal was plotted as a function of GNP concentration, ranging up to 500 ppm, to assess the detectability of GNPs dependent on the incident x-ray beam spectrum. The average XRF signals were derived from eight and four simulation sets corresponding to the x-ray dose of 1.775 and 3.55 cGy, respectively, at a given GNP concentration within the three-hole phantom. The average XRF signal versus GNP concentration was plotted for both the most favorable and the least favorable GNP detection scenarios. Then a linear fit was applied to the plotted data to obtain a calibration curve. The detection limit was defined as the lowest GNP concentration for which the XRF signals were higher than 1.96 times the standard deviation of the background (95% confidence interval)^31^. The uncertainty in the detection limit was determined by propagating uncertainties associated with the fitted calibration curve as well as the estimated background.

#### Sinogram and image reconstruction

The XRF signals from each collimator at a given rotation angle were processed to form sinogram, assigning each projection dataset denoted by *\:p*. For sinogram generation, the entire row of collimators – representing a single imaging slice – was utilized, with each collimator contributing one projection element per angle. In this dataset, each component $\:{p}_{j}$ represents the XRF signal detected by the detector corresponding to a detector collimator at a given projection angle. These projection data were then employed to reconstruct axial images of the three-hole phantom, where pixel intensities are denoted by $\:{x}_{i}$. The relationship between pixel intensities and the projection data can be expressed by the following equation,

1 $$\:{p}_{j}=\:\sum\:_{i}{M}_{i,j}{x}_{i}$$

where $\:{M}_{i,j}$ is the element of system matrix *\:M* representing the probability that the XRF signal is generated in a pixel $\:{x}_{i}$ and detected in a projection element $\:{p}_{j}$. The system matrix also incorporates a priori knowledge of linear attenuation coefficients to improve the model accuracy.

An iterative reconstruction method based on the maximum-likelihood expectation-maximization (ML-EM) algorithm^49,50^ was applied to estimate the GNP (or gold) concentration map or axial reconstructed image. Starting from an initial guess of the pixel intensities $\:{x}_{i}^{0}$, the algorithm refined these estimates through successive iterations according to the following equation,

2 $$\:{x}_{i}^{m+1}=\:\frac{{x}_{i}^{m}}{\sum\:_{j}{M}_{i,j}}\times\:\sum\:_{j}{M}_{i,j}\:\frac{{p}_{j}}{\sum\:_{i}{M}_{i,j}{x}_{i}^{m}}$$

The image reconstruction scripts based on the ML-EM algorithm were developed in MATLAB (version R2023b) and employed to generate the reconstructed images presented in the Result section.

#### XRF Signal to compton background ratio

To assess the detectability of gold K-shell XRF signals, the XRF signal to Compton background ratio (SBR) was calculated using the following equation,

3 $$\:\mathrm{S}\mathrm{B}\mathrm{R}=\frac{{\mathrm{N}}_{\mathrm{X}\mathrm{R}\mathrm{F}}}{{\mathrm{N}}_{\mathrm{C}\mathrm{o}\mathrm{m}\mathrm{p}\mathrm{t}\mathrm{o}\mathrm{n}}}$$

where $\:{\mathrm{N}}_{\mathrm{X}\mathrm{R}\mathrm{F}}$ represents the net gold K-shell XRF counts obtained by subtracting the estimated Compton background ($\:{\mathrm{N}}_{\mathrm{C}\mathrm{o}\mathrm{m}\mathrm{p}\mathrm{t}\mathrm{o}\mathrm{n}}$) from the total counts over an energy region encompassing the gold K-shell XRF peaks. This ratio (SBR) serves as a dose-normalized metric because both the $\:{\mathrm{N}}_{\mathrm{X}\mathrm{R}\mathrm{F}}$ and $\:{\mathrm{N}}_{\mathrm{C}\mathrm{o}\mathrm{m}\mathrm{p}\mathrm{t}\mathrm{o}\mathrm{n}}$ scale similarly with the deposited dose and are equally influenced by the factors such as incident x-ray beam spectrum and attenuation along the source-isocenter (phantom)-detector pathway. By evaluating the SBR across different incident x-ray spectra including the Sn-filtered 125 kVp beam and three monochromatic beams at 85, 90, and 95 keV, insights were gained into how the beam quality/spectrum affects an interplay between gold K-shell XRF signal and Compton scatter, facilitating the optimization of an incident beam spectrum for benchtop XFCT.

#### Contrast-to-noise ratio

For quantitative analysis of XFCT images, a circular region of interest (ROI) of 10 × 10-pixel area was defined within each GNP-containing insert using 16-bit grayscale images in the MATLAB software (Version R2023b). A contrast-to-noise ratio (CNR) metric was calculated using the following formula,

4 $$\:CNR=\:\frac{{S}_{ROI}-{S}_{BKG}}{{\sigma\:}_{BKG}}$$

where $\:{S}_{ROI}$ and $\:{S}_{BKG}$ represent the mean pixel values within the ROI of GNP-containing insert and a background region (located at the center of the phantom and assumed to be GNP-free), respectively. The term $\:{\sigma\:}_{BKG}$ is the standard deviation of pixel values within the background ROI, serving as a measure of noise. In accordance with the Rose criterion^51^, a minimum CNR of approximately 3 to 5 is required for reliable object detectability in imaging systems.

## Results

### Validation of the MC model

**Fig. 4:**
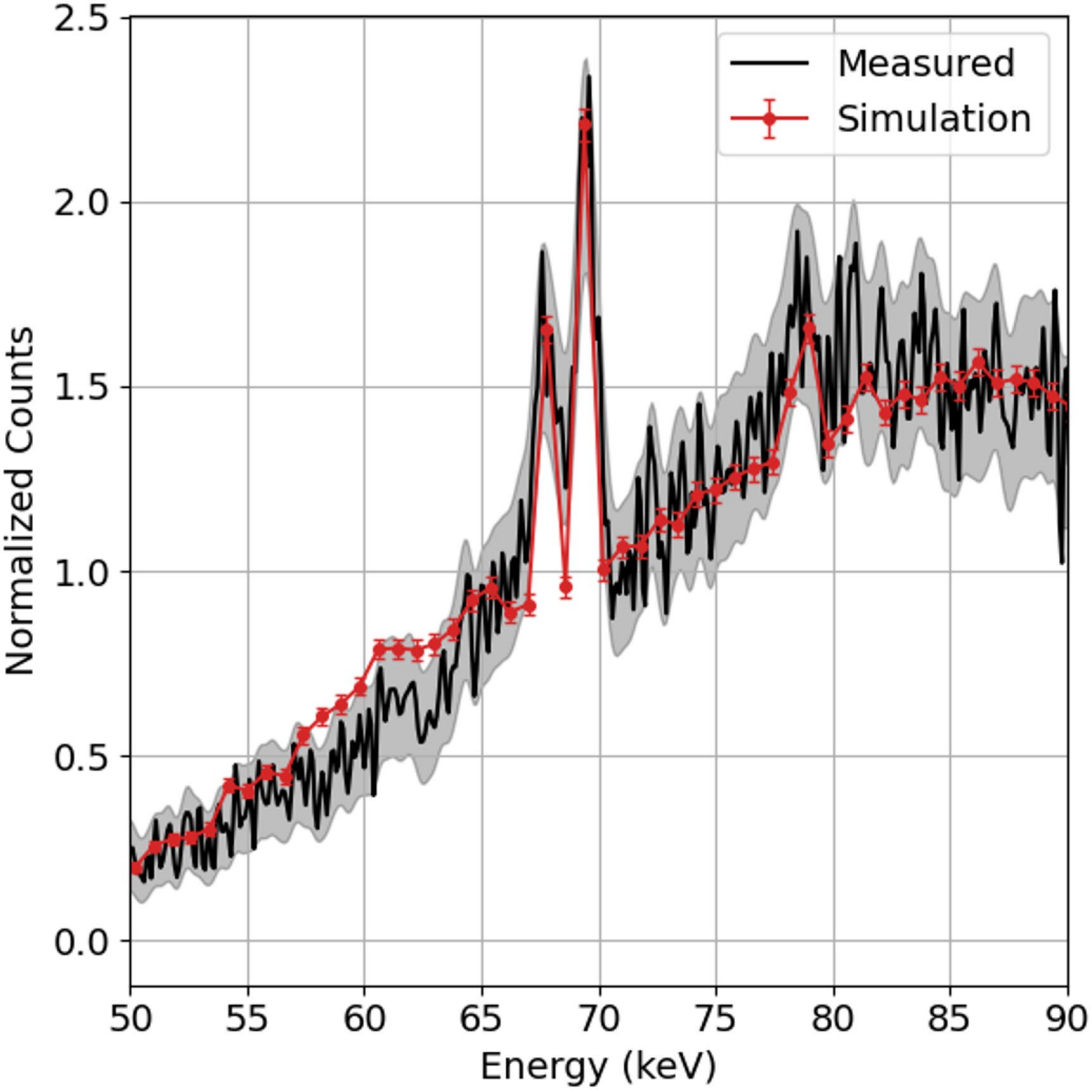
Comparison between measured and MC-simulated XRF/scatter spectra for validation of the MC model under the same condition (1.8 mm Sn-filtered 125 kVp cone-beam irradiation of a three-hole phantom containing 10,000 ppm GNP solution in one of the holes under the most favorable scenario, i.e., at rotation angle 0^o^). Both spectra are normalized to aid qualitative assessment. The MC-simulated spectrum was generated using an energy bin width of 0.8 keV. The gold XRF peaks of interest are located at ~ 67 and ~ 69 keV. The shaded band for the measured spectrum shows the uncertainty band representing the ± 1.96 σ (95% confidence) statistical uncertainty.

Figure 4 shows the comparison between the experimentally measured spectrum acquired with 10 s acquisition time and the corresponding MC-simulated spectrum with depositing an equivalent dose of 3.55 cGy at the center of the three-hole phantom containing 10,000 ppm GNP solution in one of the holes under the most favorable scenario (at rotation angle 0^o^). The measured spectrum is shown with a shaded uncertainty band representing the ± 1.96 σ (95% confidence) statistical uncertainty (assuming Poisson counting statistics) and smoothed for visualization. The calculated Pearson product-moment correlation coefficient (*r* = 0.9633) indicated excellent agreement between the MC-simulated and measured spectra.

### Calibration curves and GNP detection limits

**Fig. 5:**
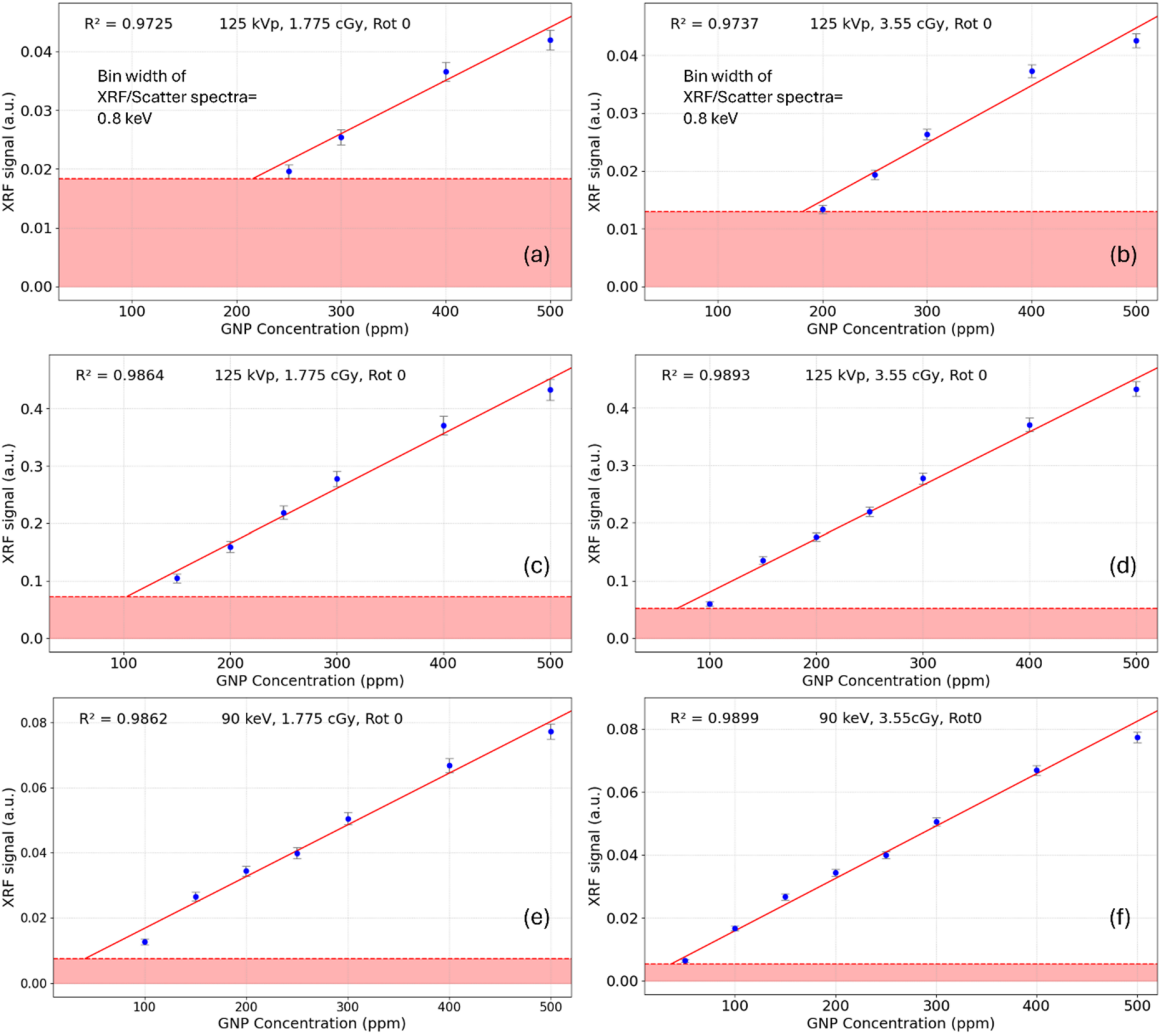
Calibration curves for the most favorable scenario of GNP detection, where the GNP-loaded hole of the three-hole phantom directly faced the detector. The corrected XRF signal is plotted as a function of GNP concentration under four irradiation conditions: Sn-filtered 125 kVp and monochromatic 90 keV beams evaluated at doses of 1.775 and 3.55 cGy. For the Sn-filtered 125 kVp beam, results are shown based on XRF/scatter spectral bin widths of 0.8 keV in panels a-b and bin widths of 0.1 keV in panels c-d, while the monochromatic beam results were analyzed using a bin width of 0.1 keV (panels e-f). The uncertainties were estimated from the statistical variations obtained by performing multiple independent simulations for each condition. All four curves show a high degree of linearity, confirming that the corrected XRF signal is directly proportional to GNP concentration across the GNP concentration range. The shaded area represents XRF signal less than 1.96 times the standard deviation of the background (noise level). Calibration curves for additional irradiation conditions (including least favorable scenario) are not shown, yet they demonstrate the same linearity.

Calibration curves were generated by plotting the corrected XRF signal - after applying the background subtraction and attenuation correction - against GNP concentration for both the Sn-filtered 125 kVp beam and monochromatic beams. These curves were constructed for both the most favorable detection geometry (phantom rotation angle 0^o^), and the least favorable detection geometry (phantom rotation angle 180^o^). In all cases involving different beam energies and dose levels, the XRF signal exhibited a strong linear correlation with respect to GNP concentrations (R^2^ > 0.96). This consistent linearity across different conditions demonstrates the XFCT system’s ability for accurate GNP quantification. Figure 5 shows the representative calibration curves for the most favorable detection geometry under four irradiation conditions including the 125 kVp and monochromatic 90 keV beams delivering doses of 1.775 cGy and 3.55 cGy. In all cases, the corrected XRF signal demonstrated a linearly proportional relationship with GNP concentration, over the comparatively lower GNP concentration range (0 to 500 ppm), affirming the consistency and reliability of XRF signal quantification across the dose levels and incident x-ray spectra considered in this study.

In addition to evaluating the linearity of corrected XRF signal with respect to GNP concentration, the sensitivity of the benchtop XFCT system was assessed by estimating the GNP detection limits. These limits were determined from the calibration curve as described in Methods. Table 2 summarizes the range of the GNP detection limits (in ppm) across different beam types and two dose levels (1.775 and 3.55 cGy) for both the most and least favorable GNP detection scenarios. Enhanced GNP detection limits were generally achieved under the most favorable geometry and at the higher dose level of 3.55 cGy. The Sn-filtered 125 kVp beam yielded the GNP detection limit of 104–140 ppm at a dose of 1.775 cGy, which improved to 69–88 ppm at a dose of 3.55 cGy. When the XRF/scatter spectrum under the Sn-filtered 125 kVp beam irradiation scenario was rearranged with an energy bin width of 0.8 keV to mimic that acquired with realistic CdTe detectors^6,33^, the estimated GNP detection limits increased, reaching 216–243 ppm at 1.775 cGy and 181–220 ppm at 3.55 cGy. While degraded from the results corresponding to an ideal detector, these GNP detection limits were still generally better (i.e., lower) than those determined experimentally (e.g., 300–500 ppm). This suggests that, unless the detector energy resolution was further improved, they could be considered as the thresholds for detection/imaging of GNPs present within small animal sized objects using the K-shell XFCT mode of our experimental benchtop cone-beam XFCT system adopting CdTe detectors. For the 85 keV monochromatic incident x-ray beam, the GNP detection limit improved from 34–70 ppm at 1.775 cGy to 32–46 ppm at 3.55 cGy. For the 90 keV monochromatic incident x-ray beam, the GNP detection limit improved from 41–117 ppm at 1.775 cGy to 37–82 ppm at 3.55 cGy. At a dose level of 1.775 cGy, the use of a monochromatic 85 keV beam resulted in up to a three-fold improvement in the GNP detection limit compared to the Sn-filtered 125 kVp beam, i.e., from 104–140  ppm to 34–70 ppm. Similarly, the monochromatic 90 keV beam improved the GNP detection limit by approximately up to 2.5-fold compared to the Sn-filtered 125 kVp beam, lowering the range to 41–117 ppm.

**Table 2:** Estimated GNP detection limits (in ppm) for various x-ray beam types and two dose levels (1.775 and 3.55 cGy). For each dose level, the two reported values correspond to detection limits from the most favorable scenario (phantom rotation angle 0^o^) and the least favorable scenario (phantom rotation angle 180^o^). For the Sn-filtered 125 kVp beam, results are additionally shown for an increased energy bin size of 0.8 keV to evaluate the impact of an energy bin size on the detection sensitivity.

Beam Type	GNP detection limit (in ppm)	
	With Dose 1.775 cGy	With Dose 3.55 cGy
Sn-filtered 125 kVp	(104 ± 5, 140 ± 7)	(69 ± 4, 88 ± 4)
Sn-filtered 125 kVp (bin size 0.8 keV)	(216 ± 12, 243 ± 16)	(181 ± 10, 220 ± 11)
85 keV	(34 ± 2, 70 ± 4)	(32 ± 2, 46 ± 2)
90 keV	(41 ± 2, 117 ± 6)	(37 ± 2, 82 ± 5)
95 keV	(70 ± 4, 120 ± 6)	(51 ± 3, 99 ± 5)

**Fig. 6:**
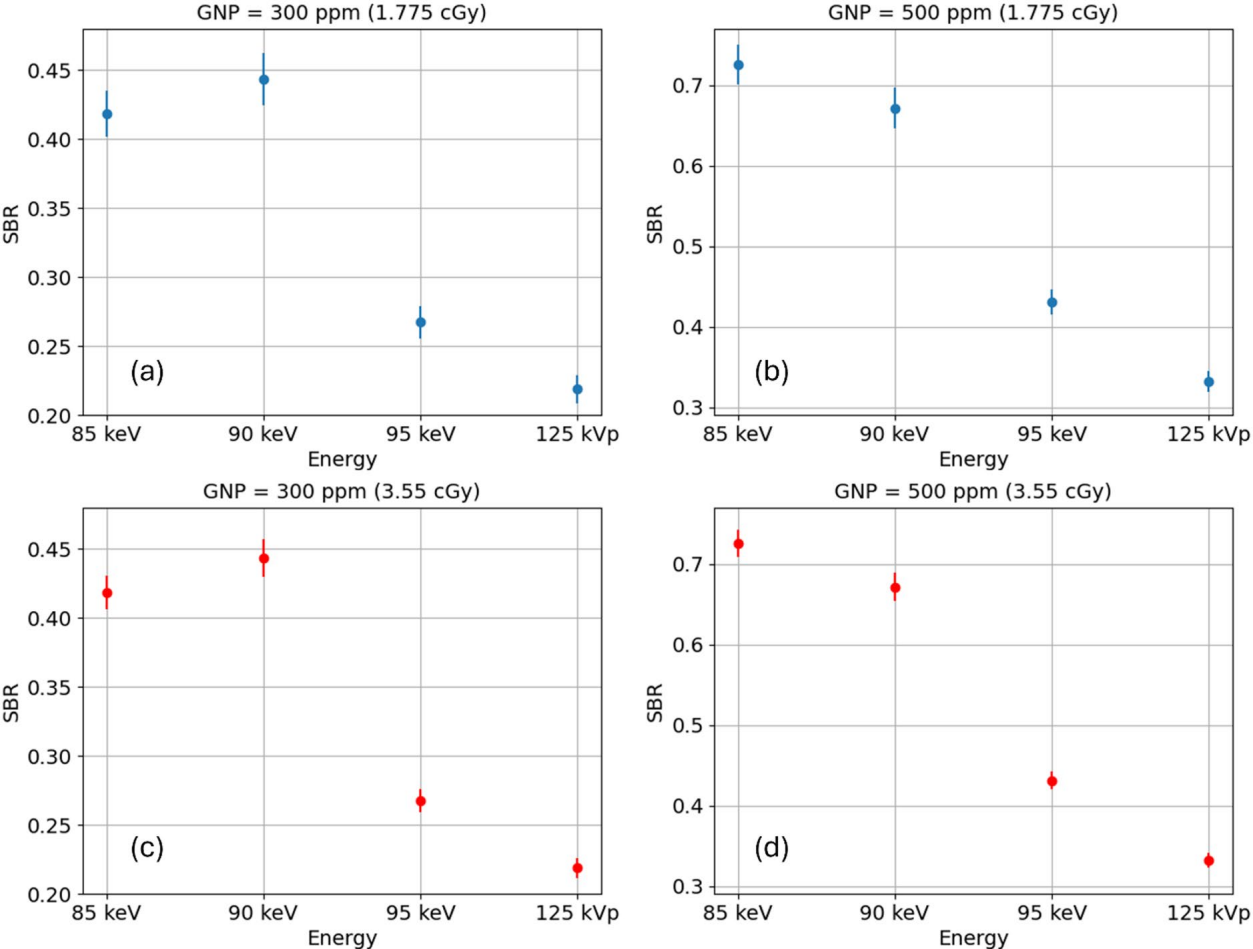
XRF Signal to Compton background ratio (SBR) as a function of incident x-ray energy – 85 keV, 90 keV, 95 keV, and Sn-filtered 125 kVp – for four irradiation and GNP concentration combinations: (**a**) 300 ppm GNP at 1.775 cGy, (**b**) 500 ppm GNP at 1.775 cGy, (**c**) 300 ppm at 3.55 cGy, and (d) 500 ppm at 3.55 cGy. The uncertainties were derived from variations among multiple independent MC simulations under the same conditions.

Figure 6 shows the SBR values corresponding to four different combinations of GNP concentration and x-ray dose: (a) 300 ppm at 1.775 cGy, (b) 500 ppm at 1.775 cGy, (c) 300 ppm at 3.55 cGy, and (d) 500 ppm at 3.55 cGy. In all cases, the SBR improved with increasing GNP concentration, suggesting enhanced XRF signal relative to the Compton background. However, it stayed almost independent of x-ray dose as expected, although its statistical uncertainty decreased with increasing x-ray dose. For each x-ray dose and GNP concentration combination, the monochromatic 85 and 90 keV beams consistently yielded higher (2 to 2.5 times) SBR values compared to the Sn-filtered 125 kVp beam. In contrast, the monochromatic 95 keV beam showed only a moderate improvement in SBR over the Sn-filtered 125 kVp beam.

### Contrast-to-noise ratios in reconstructed XFCT images

**Fig. 7:**
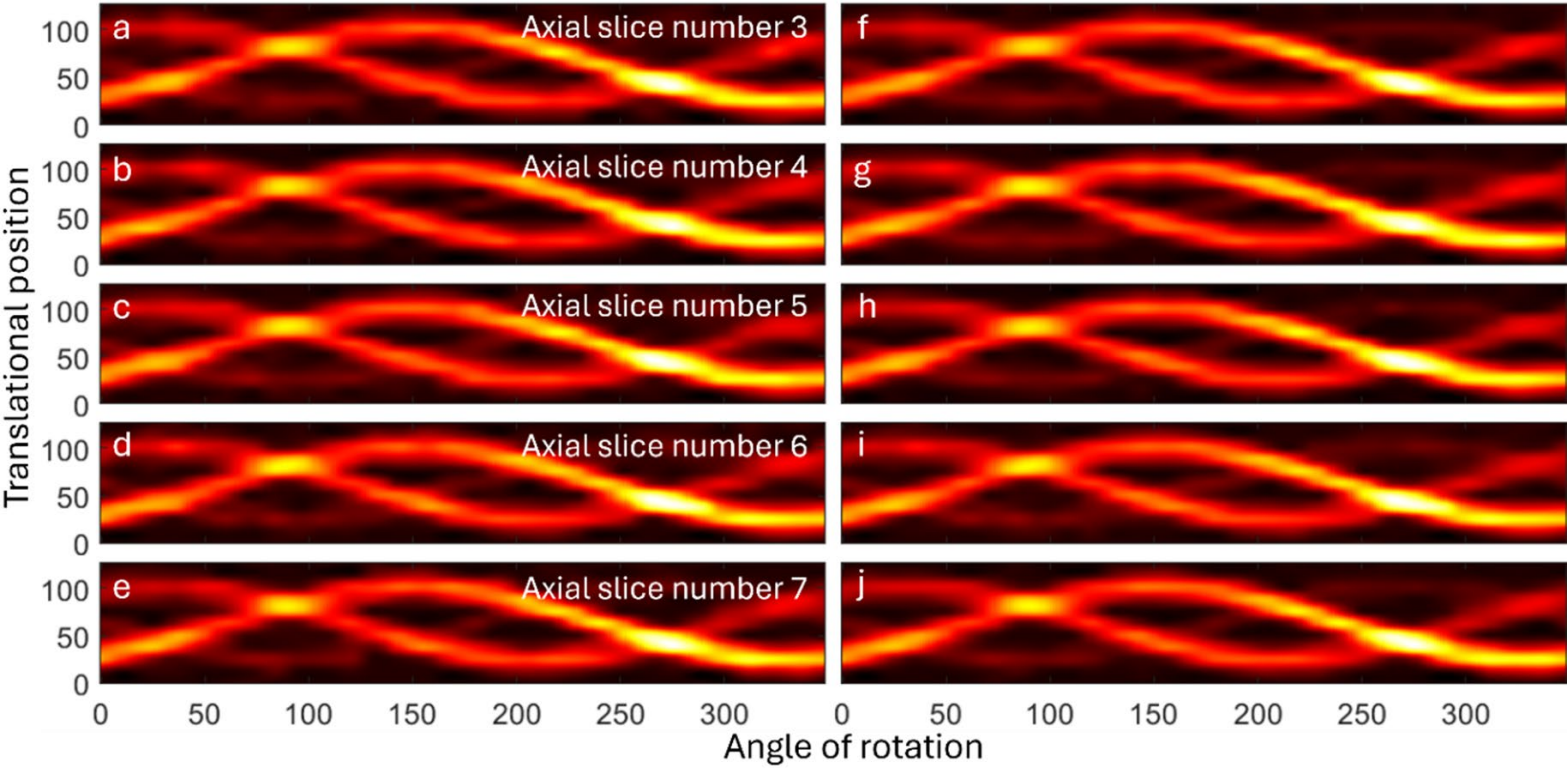
Sinograms were obtained for slice number 3 to 7 from the three-hole phantom containing GNPs at concentrations of 1000, 3000, and 5000 ppm irradiated with the Sn-filtered 125 kVp x-ray beam. Panels (**a-e**) correspond to a lower x-ray dose of 1.775 cGy, while panels (**f-j**) correspond to a higher x-ray dose of 3.55 cGy. The x axis represents the projection angle in degrees and y axis indicates the number of detector elements.

Figure 7 shows sinograms from five axial slices (slices 3 to 7 of the three-hole phantom, numbered from top to bottom ) acquired with the x-ray dose of 1.775 and 3.55 cGy using the Sn-filtered 125 kVp beam, when the three-hole phantom was filled with GNPs at concentrations of 1000, 3000, and 5000 ppm, consistent with our previous experimental work^7,35^. The sinograms on the left (Fig. 7a-e) correspond to a lower dose (1.775 cGy) case, while those on the right (Fig. 7f-j) represent a higher dose (3.55 cGy) case. Similar to the observations made from our experimental work^7,35^, the advantage of using the higher dose was evident across all five slices, with visually noticeable (qualitatively reduced) noise and enhanced XRF signal in the sinograms.

**Fig. 8:**
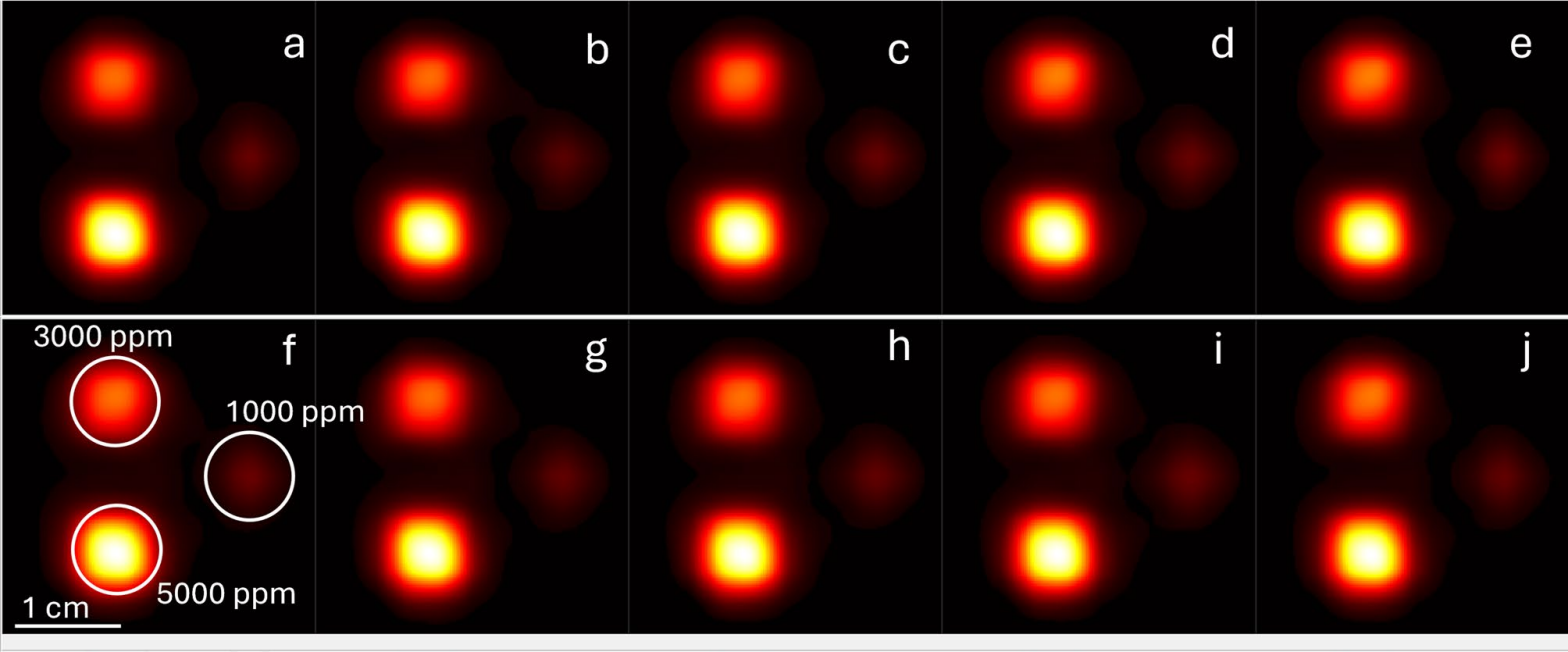
Reconstructed axial XFCT images for five slices of the three-hole phantom containing GNP concentrations of 1000, 3000, and 5000 ppm, derived from the corresponding sinograms using the iterative ML-EM method. The top row (**a-e**) shows images based on the x-ray dose of 1.775 cGy, while bottom row (**f-j**) corresponds to the x-ray dose of 3.55 cGy. The white circles in panel (f) indicate the locations of GNP-containing holes with the noted GNP concentrations.

Figure 8 shows the reconstructed XFCT images from sinograms shown in Fig. 7 by applying the iterative ML-EM method, corresponding to a lower dose of 1.775 cGy (a-e) and a higher dose of 3.55 cGy (f-j). For improved visualization, the reconstructed axial slices were interpolated by using cubic interpolation. The calculated CNR ratio for each image (Table 3) fulfilled the Rose criterion, suggesting the detectability of GNP-loaded regions. For 1.775 cGy dose, the average CNR values across all axial slices were 13.2 ± 1.5, 70.4 ± 1.6 and 120.9 ± 1.5 for GNP concentrations of 1000, 3000, and 5000 ppm, respectively. At the higher dose of 3.55 cGy, the average CNR values improved to 18.5 ± 1.1, 96.5 ± 0.90 and 164.9 ± 1.0 for the same GNP concentrations. These results highlight the benefit of increased dose (i.e., longer scan time) in achieving better image quality and more reliable detection of GNP distributions. These results align well qualitatively with those from our previous experimental study^7,35^, although the CNR values represent theoretically the best case scenario that may be achievable with an ideal detector.

**Table 3:** The CNR of the hole regions of the three-hole phantom containing GNP concentrations of 1000, 3000, and 5000 ppm in the reconstructed images at dose depositions of 1.775 and 3.55 cGy.

GNP concentrations (ppm)	Contrast-to-Noise Ratio (CNR)	
	1.775 cGy dose	3.55 cGy dose
1000	13.2 ± 1.5	18.5 ± 1.1
3000	70.4 ± 1.6	96.5 ± 0.9
5000	120.9 ± 1.5	164.9 ± 1.0

**Fig. 9:**
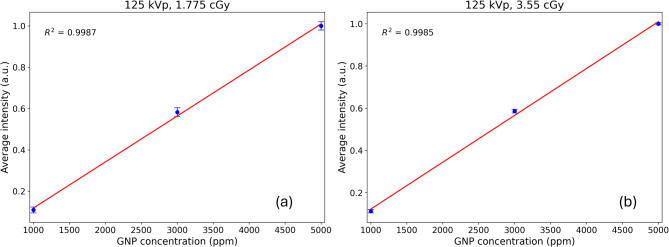
Correlation between average signal intensity estimated from reconstructed XFCT images and GNP concentrations (**a**) for the lower dose (1.775 cGy) case and (**b**) for the higher (3.55 cGy) dose case. High degree of linearity is observed in both cases (R^2^ > 0.99).

Figure 9 shows plots demonstrating the quantitative correlation between the average signal intensity from the reconstructed XFCT images and the corresponding GNP concentrations of 1000, 3000, and 5000 ppm for the two dose levels of 1.775 cGy (panel a) and 3.55 cGy (panel b). In both cases, a high degree of linearity was observed, enabling an accurate estimation of GNP concentration from the reconstructed image.

**Fig. 10:**
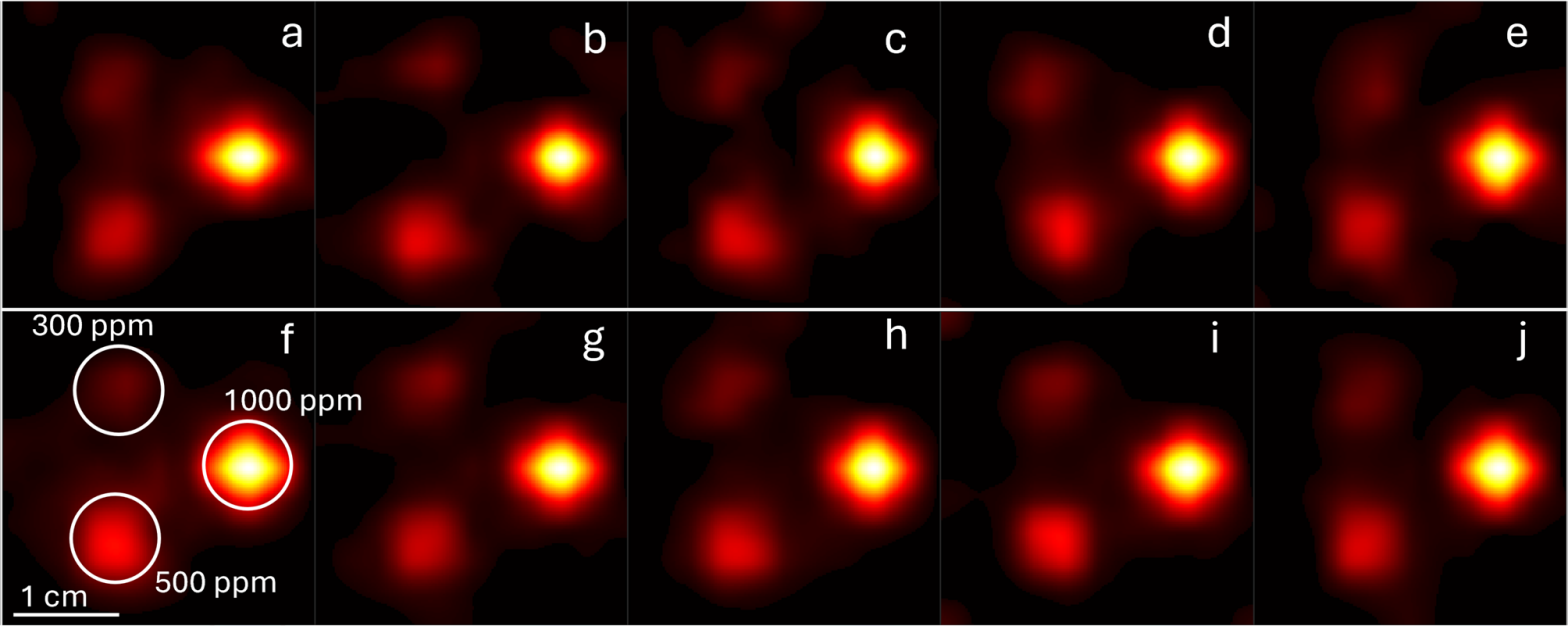
Reconstructed axial XFCT images for five slices of the three-hole phantom containing GNP concentrations of 300, 500, and 1000 ppm, derived from the corresponding sinograms using the iterative ML-EM method. The top row (**a-e**) shows images based on the dose deposition of 1.775 cGy, while bottom row (**f-j**) represents those based on the dose deposition of 3.55 cGy. In panel (f), the GNP-filled hole locations and GNP concentrations are illustrated for a reference.

Figure 10 shows the reconstructed XFCT images obtained from five slices of the three-hole phantom filled with GNP concentrations of 300, 500, and 1000 ppm. The XFCT images were reconstructed from corresponding sinograms using the iterative ML-EM method. The top row (panels a-e) represents the reconstructed images corresponding to a lower dose (i.e., 1.775 cGy) case, whereas the bottom row (panels f-j) shows the reconstructed images corresponding to a higher dose (3.55 cGy) case. As illustrated by white circles in panel (f), the locations of three GNP-filled holes and respective GNP concentrations were distinctly identified. Comparing the two sets of images demonstrates the clear effects of increased dose deposition (i.e., delivering higher dose of 3.55 cGy), such as enhanced image quality and reduced noise levels in the reconstructed XFCT images. For the lower dose (1.775 cGy) case, the average CNR values for the three GNP-filled regions (300, 500, and 1000 ppm) in the images were 4.9 ± 2.2, 6.8 ± 1.8, and 12.3 ± 2.1. For the higher dose (3.55 cGy) case, the CNR values for the same GNP-filled regions were 5.3 ± 1.6, 8.9 ± 1.1, and 17.2 ± 1.5, as summarized in Table 4. The comparison of the CNR values between the two cases quantitatively showed the effects of higher dose on the reconstructed XFCT images, similar to those shown qualitatively from comparison of the two sets of images. On the other hand, such dose-dependent effects in the CNR values appeared to be diminishing as the GNP concentration in each hole approached the detection limit (e.g., 300 ppm).

**Table 4:** The CNR of the hole regions of the three-hole phantom containing GNP concentrations of 300, 500, and 1000 ppm in the reconstructed images at dose depositions of 1.775 and 3.55 cGy.

GNP concentrations (ppm)	Contrast-to-Noise Ratio (CNR)	
	1.775 cGy dose	3.55 cGy dose
300	4.9 ± 2.2	5.3 ± 1.6
500	6.8 ± 1.8	8.9 ± 1.1
1000	12.3 ± 2.1	17.2 ± 1.5

**Fig. 11:**
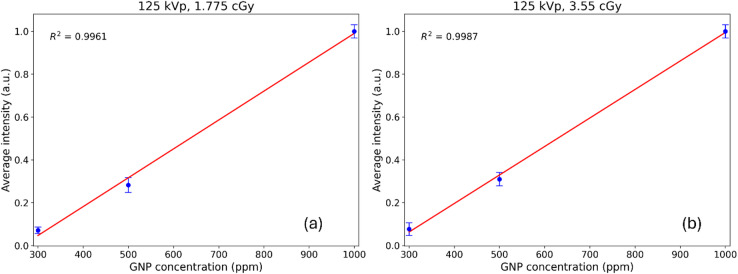
Correlation between average signal intensity estimated from reconstructed XFCT images and GNP concentrations (**a**) for the lower dose (1.775 cGy) case and (**b**) for the higher (3.55 cGy) dose case. High degree of linearity is observed in both cases (R^2^ > 0.99).

Figure 11 shows plots demonstrating the quantitative correlation between the average signal intensity from the reconstructed XFCT images and the corresponding GNP concentrations (300 to 1000 ppm) for the two dose levels of 1.775 cGy (panel a) and 3.55 cGy (panel b) with the Sn-filtered 125 kVp beam. In both cases, a high degree of linearity was observed (R^2^ > 0.99), confirming the robustness of XFCT for quantitative GNP imaging.

## Discussions

This study successfully developed a Geant4-based MC model of an experimental benchtop cone-beam XFCT system employing a larger FOV pixelated CdTe detector, properly reflecting an anticipated detector upgrade for our latest experimental benchtop XFCT setup^7^. This MC model effectively simulated both hypothetical and realistic K-shell XFCT imaging scenarios involving a GNP-containing three-hole phantom under four different x-ray beam irradiation conditions. For the cases with the Sn-filtered 125 kVp beam, direct comparisons between the results from the current MC study and our previous experimental investigations^7,31,35^ were possible, providing valuable insight into potential thresholds for the performance of our experimental benchtop cone-beam XFCT system. For example, given the current MC results based on the 0.8 keV energy bin size (Table 2), both levels of doses considered in this study (i.e., 1.775 and 3.55 cGy) would allow for the detection/imaging of GNPs present at even lower concentrations (e.g., 250 ppm) within small animal-sized objects (e.g., the three-hole phantom in this study) than those considered in our previous experimental studies. Thus, future research efforts may be directed towards investigating the factors that are difficult to incorporate during the MC modeling but may affect the experimental detectability of GNPs at such low concentrations (e.g., factors affecting the detector performance^6^. If successful, such efforts will allow for our existing benchtop cone-beam XFCT system (as well as an upgraded version with a larger FOV detector) to achieve the GNP detection limit close to the level as projected in the current MC study. Moreover, as demonstrated in our previous investigations^33,52,53^, further performance enhancement of our existing benchtop cone-beam XFCT system will also be possible through additional hardware optimization (e.g., further optimization of the detector collimator) and more advanced spectral data handling techniques (e.g., deep learning-based XRF signal extraction and XFCT image reconstruction). Taking the aforementioned efforts and approaches altogether, our future benchtop cone-beam XFCT system may achieve the GNP detection limit close to the level previously suggested as the GNP detection threshold (~ 100 ppm) for benchtop K-shell XFCT systems^30^ under the experimental conditions similar to those considered in this study.

While subject to further optimization efforts including the detector upgrade as noted above for a wider range of applications, our existing benchtop cone-beam XFCT system is considered acceptable for in vivo imaging within the scope of many practical applications. For example, under the K-shell XFCT mode and in conjunction with transmission CT in the same platform^31^, this system can be used for the determination of passively targeted (unconjugated) GNP biodistribution in live mice during preclinical studies of GNP-enhanced radiotherapy that involve relatively larger amounts of GNPs across the xenograft tumor and critical organs (e.g., GNP concentration of ~ 5000 ppm)^23,28,54^, while closely or better meeting the x-ray dose and scan time requirements as suggested for the whole body in vivo imaging of GNP-containing mice^30^. As necessary, given the currently achievable GNP detection limit at a given dose (e.g., 500 ppm at 1.775 cGy per projection) and the aforementioned level of GNP concentration during preclinical studies of GNP-enhanced radiotherapy, the x-ray dose/scan time for K-shell XFCT of GNP-containing mice can be further reduced drastically (e.g., by one order of magnitude or more). The image resolution achievable from our existing benchtop cone-beam XFCT system (~ 2.8 mm^7^) is also considered sufficient for the determination of GNP biodistributions during such preclinical studies, given the typical size of xenograft tumors and critical organs. Further improvement in the image resolution (e.g., closely matching the 2 mm detector collimator aperture size) is also possible from applying more sophisticated XFCT image reconstruction algorithms that we developed^53,55^. Overall, the technical specifications summarized above have been confirmed through our unpublished work where we performed multiple times an in vivo version of our postmortem benchtop XFCT imaging study of tumor-bearing mice injected with GNPs^8^. Besides its usage for GNP-enhanced radiotherapy applications, the K-shell XFCT mode of our existing or upgraded system can also be used for the same purpose during preclinical testing of other high-Z MNPs such as HfO_2_ NPs for radiotherapy enhancement^30,56^.

For the monochromatic x-ray beam cases, the current MC results, although they cannot be compared with experimental data at this time, may still serve as reasonable guidance for future investigations, given the validity of our MC model. From a physics point of view, the incident x-ray beam spectrum is the main factor that affects the sensitivity (material detection limit) of a benchtop XFCT system. In our previous MC study^36^, we showed that the use of hypothetical quasi-monochromatic x-ray beams in our original benchtop cone-beam XFCT system could improve the net XRF signal (extracted from the acquired XRF/scatter spectra and directly related with the system sensitivity) per a given dose by more than one order of magnitude. A similar observation was also made by a different group^37^ that investigated a strategy to improve the sensitivity of a benchtop XFCT system by using monochromatic incident beams in combination with further angulation of an XRF detector. Since various conditions modeled in these two computational studies were not identical (e.g., cone-beam vs. pencil-beam for the incident beam geometry), quantitative findings from them cannot be generalized. Nevertheless, both studies confirmed the importance of incident x-ray beam spectrum optimization for further enhancement of the sensitivity of a benchtop XFCT system while demonstrating the possibility of such drastic improvement in the system sensitivity, if technically feasible, by adopting monochromatic (or quasi-monochromatic) x-ray sources. In the current MC study, thus, we also performed a quantitative investigation of this possibility by replacing the Sn-filtered 125 kVp beam with three hypothetical monochromatic x-ray beams (85, 90, and 95 keV) for more favorable gold XRF production and detection.

As detailed in the Result section, analysis of the XRF signal-to-Compton background ratio highlighted the superior performance of monochromatic x-ray beams, particularly at 85 and 90 keV, compared to the Sn-filtered 125 kVp beam adopted in our recent two benchtop XFCT systems^7,31^. This improvement is attributed to the higher gold K-shell XRF photon yields with monochromatic x-ray beams having their energies above the gold K-edge (80.7 keV) than those associated with polychromatic x-ray beams, in combination with the decreased Compton scatter background around the gold K-shell XRF peaks by such monochromatic incident x-ray beams^36,37,57^. Overall, according to the current MC results, while not as great as the level predicted in our previous study^36^, the sensitivity improvement was remarkable (e.g., up to more than three-fold). The noted difference in the predicted sensitivity improvement between the two studies can be attributed mainly to the differences in MC modeling parameters and the study scope. For example, our previous MC model^36^ was based on our early experimental benchtop XFCT setup adopting a microfocus 105 kVp x-ray source and a single crystal CdTe detector^12,32^. In addition, our previous MC study^36^ focused only on investigating the effects of incident beam spectra on the XRF production/detection when the three-hole phantom was filled with GNPs at a much higher concentration (i.e., 20,000 ppm or 2 wt%) than that considered in the current MC study (e.g., lower by almost two orders of magnitude). As a result, the quantitative observations made from both studies could not be identical. In fact, the main focus of the current MC study was to investigate the detection/imaging of GNPs present within the three-hole phantom (or small animal-sized objects) at concentrations close to the detection limit as determined from our recent experimental study^7,35^.

While strongly suggesting the possibility of considerable improvement in the GNP detection limit beyond the currently achievable level, adopting monochromatic/synchrotron x-ray beams for K-shell XFCT on a benchtop setting is currently not feasible, to the best of our knowledge. As noted in our previous MC study^36^, however, crystal-based approaches based on Bragg diffraction, such as the use of highly oriented pyrolytic graphite (HOPG) crystals^58^, may still be pursued to generate quasi-monochromatic beams suitable for K-shell XFCT, despite some known limitations, such as significant loss in the incident beam intensity and requirement of very shallow beam incident angles to produce x-rays with energy above the gold K-edge. For further research into this direction, it is worth noting that similar crystal-based approaches have been shown successful for production of quasi-monochromatic x-ray beams up to ~ 60 keV energy for radiotherapy purposes^59,60^. Besides crystal-based approaches, the incident x-ray beam spectrum for our experimental benchtop XFCT system can be optimized further by a judicious combination of metal filter and x-ray tube potential (kVp). In fact, while not investigated in this MC study, our previous MC study^36^ suggested continued improvement in the net XRF signal per a given dose by further hardening of the polychromatic incident x-ray spectrum. Thus, it is conceivable in principle to use higher kVp and/or thicker Sn-filter for the K-shell XFCT mode of our experimental benchtop cone-beam XFCT system, as long as it does not cause any serious negative consequences (e.g., prolonged scan time, heavier radiation shielding, etc.). In our future study, the approaches mentioned above will be investigated in detail to assess their potential merits and feasibility.

## Conclusion

This work demonstrated that a new MC model of our experimental benchtop cone-beam XFCT system could accurately replicate experimental performance under the K-shell XFCT mode. The validated MC model showed that GNP detection limits of approximately 104 ppm at 1.775 cGy and 69 ppm at 3.55 cGy could be achievable during the irradiation of a small animal-sized GNP-containing three-hole phantom with the Sn-filtered 125 kVp beam, under the ideal detection scenario. According to the MC results, the adoption of monochromatic beams at energies just above the gold K-edge significantly enhanced the system sensitivity, improving the GNP detection limits by up to three-folds at a given dose (e.g., 34–70 ppm at 1.775 cGy with the 85 keV beam). This improvement can be attributed to both increased XRF photon yield and reduced Compton background when such monochromatic beams are adopted as incident beams for K-shell XFCT. Although monochromatic beam sources are currently impractical for benchtop XFCT systems, the results of this work highlight the importance of beam spectrum optimization, including the use of quasi-monochromatic beams or further filtration of polychromatic beams (leading to further hardening of the beam spectra), to improve the sensitivity of K-shell XFCT. Overall, this MC study provided theoretical limits for detecting/imaging GNPs present within small animal-sized objects using K-shell XFCT, setting benchmarks for future research and development of benchtop XFCT systems as well as preclinical imaging applications.

## Data Availability

The data that support the findings of this study are available from the corresponding author upon reasonable request.
